# A prospective, multi-method, multi-disciplinary, multi-level, collaborative, social-organisational design for researching health sector accreditation [LP0560737]

**DOI:** 10.1186/1472-6963-6-113

**Published:** 2006-09-12

**Authors:** Jeffrey Braithwaite, Johanna Westbrook, Marjorie Pawsey, David Greenfield, Justine Naylor, Rick Iedema, Bill Runciman, Sally Redman, Christine Jorm, Maureen Robinson, Sally Nathan, Robert Gibberd

**Affiliations:** 1 Centre for Clinical Governance Research, Faculty of Medicine, University of New South Wales, 10 Arthur St, Kensington, NSW 2052, Australia; 2 School of Public Health and Community Medicine, Faculty of Medicine, University of New South Wales, Samuels Building, Kensington, NSW 2052, Australia; 3 Australian Council on Healthcare Standards, 5 Macarthur Street, Ultimo, NSW 2007, Australia; 4 Centre for Health Informatics, Faculty of Medicine, University of New South Wales, Cliffbrook Campus, University of New South Wales, 45 Beach Street, Coogee 2034, Australia; 5 Australian Patient Safety Foundation, c/- Royal Adelaide Hospital, GPO Box 400, Adelaide, South Australia 5001, Australia; 6 The Sax Institute, Building 10, Level 8, University of Technology, Sydney, 235 Jones Street, Ultimo NSW 2027, Australia; 7 The University of Newcastle, Callaghan, NSW 2308, Australia; 8 Communio Pty Ltd, PO Box 1796, North Sydney NSW 2059, Australia

## Abstract

**Background:**

Accreditation has become ubiquitous across the international health care landscape. Award of full accreditation status in health care is viewed, as it is in other sectors, as a valid indicator of high quality organisational performance. However, few studies have empirically demonstrated this assertion. The value of accreditation, therefore, remains uncertain, and this persists as a central legitimacy problem for accreditation providers, policymakers and researchers. The question arises as to how best to research the validity, impact and value of accreditation processes in health care. Most health care organisations participate in some sort of accreditation process and thus it is not possible to study its merits using a randomised controlled strategy. Further, tools and processes for accreditation and organisational performance are multifaceted.

**Methods/design:**

To understand the relationship between them a multi-method research approach is required which incorporates both quantitative and qualitative data. The generic nature of accreditation standard development and inspection within different sectors enhances the extent to which the findings of in-depth study of accreditation process in one industry can be generalised to other industries. This paper presents a research design which comprises a prospective, multi-method, multi-level, multi-disciplinary approach to assess the validity, impact and value of accreditation.

**Discussion:**

The accreditation program which assesses over 1,000 health services in Australia is used as an exemplar for testing this design. The paper proposes this design as a framework suitable for application to future international research into accreditation. Our aim is to stimulate debate on the role of accreditation and how to research it.

## Background

### Introduction

Institutional accreditation is predicated on the application of nationally and internationally agreed standards for assessing and benchmarking the performance of organisations. Typically, this involves certification by an external body, often following formalised visits by peer assessors or surveyors. The primary intent of such certification is to identify poor, satisfactory or exemplary performance. Another goal is to signal which organisations deliver products or services which are acceptable to consumers, funders and stakeholders. After decades of accreditation development in health, and multi-million euro, dollar and pound investments, the extent to which accreditation processes and outcomes accurately reflect and motivate high quality clinical and organisational performance is poorly understood and under-investigated. The need to undertake research in this area is of considerable public interest given the extent and cost of accreditation processes in use today [1-6] and the importance to consumers of efforts to improve the safety and quality of health care services [7].

This paper presents a research design which comprises a prospective, multi-method, multi-level, multi-disciplinary approach to assess the validity, impact and value of accreditation within the Australian health sector. However the generic nature of the framework makes it suitable for adoption by international researchers seeking to identify how accreditation processes influence and reflect health care organisational quality. The paper describes the process of developing the design and the rationale which underpins it.

### Accreditation as a world-wide phenomenon

Accreditation is a world-wide phenomenon [8] with large-scale investments in accreditation mechanisms in many industries and organisations. Inspection and standards-setting processes exist for industries ranging from manufacturing [9], seafood [10] and health [11], to organisations as diverse as schools [6], universities [5,12], software companies [2] and ambulance services [1]. Accreditation processes have emerged for laboratories [13], management systems [14], products [15], medical curricula [16] and staff competencies [17]. In a typical accreditation process many variables are assessed such as team and overall organisational performance, leadership, organisational culture, service or product outcomes, and customer focus. One prominent accreditation process crossing many industries is ISO 9000 [18]. It is a widely used, generic form of accreditation which concentrates on ensuring organisations have sound, documented processes to improve the way services and products satisfy customers' requirements for quality.

Essentially, accreditation processes have core normative concerns to provide barometers of performance by ensuring that organisations satisfy pre-designated standards, are regularly examined and continuously improved and embrace customer satisfaction [16]. Prevailing views suggest the benefits of accreditation processes are multiple and include: improved quality in the production of goods and services [19]; explicit, documented processes [20]; effective teamwork [21]; reduced costs [22]; and enhanced organisational cultures [23]. In other words, accreditation is advocated as a process that drives the full gamut of improvement which necessarily encompasses the structures, processes and outcomes of service delivery. Award of full accreditation status is perceived in many industries, including health, as a valid reflection of individual and organisational performance. However, few studies have empirically demonstrated this perception [19,24-27]. The value of accreditation, therefore, remains largely under-investigated and this is a significant legitimacy problem for accreditation providers, policymakers and researchers.

### The value for money problem

Accreditation in the health sector has changed continuously since its inception in the United States of America [28] in the 1950s. Australia is an instructive case study of this process. Australia was an early adopter of the accreditation concept in health and has a mature accreditation system with 76% of all hospitals, and 94% of all beds, accredited [29]. These figures reflect a major investment in accreditation in health including financial allocations, systems changes, clinical and managerial efforts, data-gathering costs, compliance measures, documentation and other commitments to designing and participating in accreditation processes [14,20,30,31]. Accreditation processes consume considerable health care resources in an environment of funding pressures. While no precise figures are available for health services expenditure on accreditation within Australia or elsewhere, various assessments show positive results from accreditation [32]. Annual costs have been estimated for a typical, medium-sized member organisation of perhaps US$630,000 per annum to participate in accreditation processes, with preparation and first year costs for an initial survey being in the order of an additional US$370,000 [33,34]. As these are costs for one health care organisation the extrapolated, whole-of-system financial commitments are likely to be very large. The significant financial costs associated with accreditation renders the need to investigate the relationships between accreditation and performance a public health priority. Thus a core question is to determine whether the process provides value for money and a sustainable return. This in turn will direct an answer to the question as to whether future investment is justified and, if so, in what ways?

### The present status of research into accreditation

While accreditation has been widely adopted both in Australia and elsewhere as a mechanism for assessing and improving health care quality [35,36], research into its effectiveness is at an embryonic stage. This is the classic lag effect between policy innovation and its subsequent research and evaluation. Moreover, existing research lacks rigorous in-depth analysis of accreditation processes and the relationships between accreditation and performance. It seems logical that such relationships hold, and many stakeholders believe this [37], but beliefs rest on attitude surveys [38], anecdotal [39], conjectural [40,41] or case study [24,30,42] evidence rather than targetted, multi-site empirical evidence. No positive or consistent relationships between accreditation and clinical performance have been found [43-45]. This does not necessarily mean that the logic upon which accreditation is based is flawed. Rather, it calls for rigorous methods with sufficient metrics to differentiate higher and lower performers and be sufficiently sensitive to detect poor clinical and organisational processes.

It is reasoned that an effective accreditation research program will not only identify poor performing areas but will be sufficiently sensitive to predict poor performance and thus help avert clinical or administrative failure. The multiple inquiries into what has gone wrong in acute settings in many countries including Australia, Canada and Britain [46] signal how important it is to have early warning systems; invoking continuous or regular accreditation processes, and providing evidence for how they contribute, are thought to be key components in addressing this.

### The research exemplar

The central organisation associated with accreditation in health in Australia is the Australian Council on Healthcare Standards (ACHS); 63% of all Australian public hospitals, and 84% of public beds, are ACHS accredited [29]. Similarly, some 74% of private acute and psychiatric hospitals are ACHS accredited [29]. The ACHS is a not-for-profit company, independent of public or private funding, whose Council comprise key stakeholders in the health industry. Apart from being the major health care accreditation body in Australia, the ACHS has an international reputation, being the third oldest health care accreditation body in the world after those of the United States and Canada.

ACHS was the first in the world to introduce clinical indicators as part of the health care accreditation process. Clinical indicators are tools used to measure dimensions of care and services. Presently, 55% of indicators measure the safety dimension and 43% measure the effectiveness dimension of health care performance [47]. Consequently, accreditation in Australia has evolved over a decade from a relatively static, standards-based endeavour to a process that incorporates various performance data in the form of clinical indicator sets [47]. More recently, the ACHS has moved accreditation into the era of continuous quality improvement as reflected in the Evaluation and Quality Improvement Program (EQuIP) [11]. This is characterised as a more holistic accreditation process focused on continuous improvement associated with increasingly stringent standards, insistence on the involvement of consumers in decision-making, and mandating that accredited organisations be seen to embrace the continuum of care [14,26]. EQuIP can be understood, therefore, to mirror, and indeed extend, developments in other quality endeavours such as ISO 9000 [9].

The ACHS, through EQuIP, conducts an advanced, well-defined and accessible accreditation process which generates extensive data in three broad forms: organisational profile information, organisational and individual performance data, and detailed text data in the form of surveyors' reports. EQuIP is designed to "guide organisations seeking accreditation through a four-year program of self-assessment, organisation-wide survey and periodic external review conducted by industry peers to meet standards" [11]. Accreditation status is conferred when an organisation demonstrates that it meets these ACHS standards.

## Methods/design

### Investigators

We assembled a team of experienced social and clinical researchers to design a program of research in order to investigate the ACHS accreditation process, outcomes and impact. In developing this program of research the team had four aims: to survey the literature; to test the initial design against a range of stakeholder groups for face validity; to incorporate into the design a comprehensive set of examinations which would provide data to inform and direct health policy regarding accreditation; and to articulate a design not only to suit an investigation of the ACHS accreditation process but which could be, with modification, used by international research groups seeking to examine other accreditation processes.

The research team comprised 12 senior investigators with expertise in organisational behaviour, organisational psychology, health quality and safety, social sciences, health informatics, health services research, statistics, health consumer needs, accreditation, and the chief areas of clinical practice (medicine, nursing and allied health). The emerging design was subject to numerous discussions, exposure drafts and re-drafts until all were satisfied as to its capacity to yield appropriate results. It was modified progressively in the light of consultations with several peak health care bodies, four industry partners in both private and public health care, the nine government jurisdictions of Australia (six States, two Territories and the Federal (Commonwealth) government, and various consumer representatives.

### Research program

**Figure 1:**
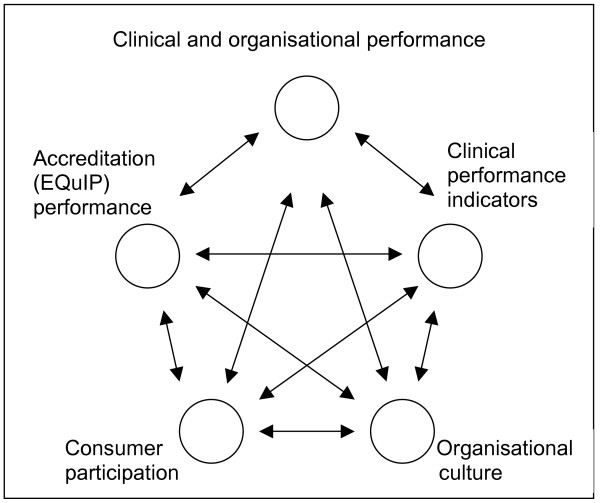
Hypothesised interrelationships between organisational characteristics.

The research program sought to investigate five major variables central to the clinical and organisational performance of an organisation (organisational performance; clinical performance indicators; organisation culture; consumer participation; and accreditation performance on EQuIP) and the inter-relationships between these variables. In Figure 1 we present a simplified model of these complex inter-relationships.

This model suggests that these characteristics are associated, and that performance of one is related to performance of another. For example, organisational performance should be directly or indirectly affected by attainment of accreditation standards (in this case, performance on EQuIP), strong clinical performance, productive involvement of consumers [48] and an effective organisational culture [49]. At this stage the nature of the relationships between all these factors is unknown. For example, does poor performance on accreditation predict poor clinical and organisational performance? If not, is the dissonance explainable?

In view of the multi-dimensional nature of health care performance, a research strategy investigating these dimensions necessarily engages both quantitative and qualitative methodologies. Dimensions not readily captured through archetypal continuous measurement tools can be caught using an array of social research tools. No other research projects were identified which have tried to investigate the relationships between accreditation and other key organisational variables using a multi-method strategy, so we had little basis on which to build a design. Thus, the strategy proposed is novel and innovative. We are unable to show causation through a randomised controlled trial (that is, between accreditation and the other variables) due to potentially confounding variables; for example, previous exposure to accreditation processes and bias due to self-selection amongst the participating and non-participating health services. Hence the necessity to examine associations rather than causality, and to use qualitative and quantitative methods, to increase our understanding of these relationships. To be useful, any research findings will need to quantify the association between accreditation and organisational and individual performances, clarify the actual and potential role of accreditation in evaluating care, and provide an evidence-base for the future development of accreditation in health and other industries. Further, an important methodological outcome will be the trialling of the multi-method research design for future research programs.

### Proposed aims and objectives

The proposed research program has two central aims addressed by six specific research objectives. We outline these in turn.

#### First research aim

The first research aim is to examine the relationships between accreditation status and processes, and the clinical performance and culture of health care organisations.

There are four proposed objectives relating to this first aim. They are as follows:

##### Research objective 1: To determine whether there is a relationship between accreditation status (as measured by EQuIP) and organisational cultural characteristics

We hypothesise that if the accreditation process is successful in improving the delivery of services through organisational change, then relative performance on EQuIP (based on standards criteria) will be associated with observable health service cultural characteristics. Thus, a health service with exemplary performance on EQuIP should exhibit positive organisational cultural features like sound relationships, positive practices, strong attitudes in favour of continuous improvement and a team-oriented approach to care.

##### Research objective 2: To assess the relationship between accreditation status and clinical performance

We hypothesise that if the accreditation process is successful in improving the standard of care, then relative performance on EQuIP should be positively associated with improvements in clinical performance. Thus an organisation with exemplary performance on the 19 mandatory EQuIP criteria should have demonstrated improvements in clinical performance, the number of care-related consumer complaints, and the number of sentinel and adverse events, or Coroner's cases which generate recommendations. We would also investigate the individual relationships between individual criterion and indicator levels, for example contrasting the infection control system with the hospital infection rate.

##### Research objective 3: To analyse the associations between consumer participation, accreditation status and organisational cultural characteristics

We hypothesise that if the accreditation process is successful in promoting participation of consumers, then relative performance on EQuIP should be positively associated with higher-level consumer participation both at individual care level and in broader governance structures. Consumers' participation in their own care has been linked with positive quality of care, treatment outcomes and reduced hospital and medical visits [50-53]. Most commentators assume that consumer participation is positively related to improved performance on standards [54,55]. However, the relationship between participation of consumers at the care level and in broader system level processes, such as in quality improvement or advisory groups, and performance on standards, have not been effectively examined.

##### Research objective 4: To evaluate the relative performance, on quality of care measures, between health services participating in and not participating in accreditation

We hypothesise that if the accreditation process is successful in improving the standard and delivery of care, then health services participating in EQuIP should demonstrate better performance on quality care measures than those which do not. The answer to this question will provide comparative evidence *vis à vis *a sample of controls – that is, those who have never participated in accreditation.

#### Second research aim

The second research aim is to examine the influence of accreditation surveyors by assessing the reliability of the accreditation process and the effect of accreditation surveyors on their own health organisations. There are two proposed objectives related to this aim. They are as follows:

##### Research objective 5: To appraise the intra- and inter-rater reliability of EQuIP surveyors and survey teams

We hypothesise that if the EQuIP instrument is reliable, performance on EQuIP should be independent of the different surveying teams. Establishing the reliability of an instrument or process is critical to understanding its limitations. EQuIP is a document-technology that requires interpretation by the surveyors. The reliability of the EQuIP instrument is potentially affected by inconsistency between surveyors. A central question therefore is whether or not different surveyors and different teams of surveyors are reliable judges of health service performance using EQuIP. The results of this investigation would have implications for how surveyors are trained and the tools needed to improve intra- and inter-rater reliability across different settings.

##### Research objective 6: To examine the relationship between accreditation status, clinical performance, organisational cultural characteristics and the number, network influence and characteristics of surveyors

We hypothesise that the presence and influence of surveyors in an organisation has a positive association with its own health service performance on EQuIP, clinical performance indicators and organisational culture. In this light, a health service with multiple surveyors would presumably benefit in measurably greater ways compared with a health service which had few or no surveyors on staff.

### Design

**Figure 2:**
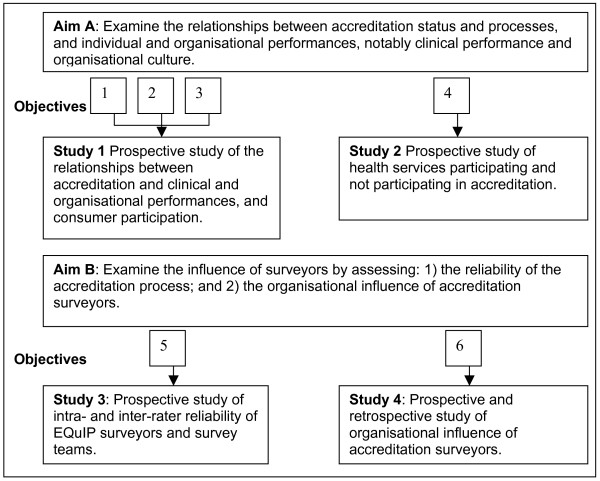
Schematic of the proposed research design.

These research objectives require a project utilising a multi-method [56] multi-level [57] approach incorporating multi-layered data [58]. In conducting the research program, a wide range of evaluation techniques need to be applied including more objective measurements, for example clinical indicator data, as well as ethnographic observations. In this way the research will investigate performance in terms of empirical data, to compare what people record, and what people say occurs, and observations of what actually occurs. The strength of this design is that it allows triangulation of results. To this end, four inter-related studies, three prospective studies and one prospective and retrospective study have been designed to meet our aims and objectives (Figure 2). The Human Research Ethics Committee of the University of New South Wales approved the project on 25 May 2005 (HREC 05081). The design features are discussed below.

#### Study 1: Prospective study of the relationships between accreditation and clinical and organisation performances, and consumer participation profiles

A random stratified sample of 20 currently accredited health services would be prospectively studied at the time of EQuIP assessment. For the measurement of clinical performance, the ACHS clinical indictor data would be independently reviewed by researchers blind to the EQuIP outcome. The EQuIP assessment incorporates submission of clinical performance data collected by health service staff. Data includes operationally defined ratios and scores for clinical indicator performance across a range of clinical areas in a specified time period.

Concurrently, but independently from the EQuIP accreditation process, each health service will be subjected to a comprehensive prospective cultural assessment. This would include direct observation and interviews targeting organisational practices, communication processes, work standardisation, and consumer participation. Previous investigations of work standardisation [59] and cultural analysis [60] provide the basis for the tools and methods to do this. This study would be grounded in ethnography, involving observation of managerial work, interviews with relevant clinician-managers and lay managers, and a survey targeting perceptions of the relevance and effectiveness of accreditation measurements as defined under EQuIP [61,62]. Other independent, standardised organisational performance data would be collected, for example number of sick days per employee, the rate of injuries to staff, staff turnover, and information about the organisational learning and development program.

Following accreditation the relationships between EQuIP performance and clinical performance and the cultural assessment would be examined. For this study, analysis would involve both quantitative and qualitative techniques. Quantitative analyses would include descriptive statistics and regression analyses. Simultaneously, and blinded to the quantitative analyses, qualitative analyses would be based on grounded theory [63] with both induction and deduction utilised to draw together the empirical data with the theoretical material.

#### Study 2: Prospective study of health services participating in and not participating in accreditation

All health services not participating in accreditation (EQuIP or otherwise) would be identified. These organisations will be matched with health services which participate in accreditation. These non-participating health services would be subjected to the same analyses as the participating health service (from study 1), that is, subjected to a comprehensive prospective cultural assessment, and subjected to the same review of performance measures. Comparison of the cultural assessments would seek to identify similarities and differences between the organisations.

#### Study 3: Prospective study of intra- and inter-rater reliability of EQuIP surveyors and survey teams

There would be three parts to this study. Firstly, an examination of survey teams in practice would be undertaken. A sample of health services currently accredited and requiring re-accreditation would be randomly selected for study. Two teams would be matched and undertake the EQuIP surveying process together for two health services. During the surveying process the two teams would independently undertake interviews with relevant health service staff. The genuine accrediting team would be concealed from both the surveyors and health service. The teams would be asked to keep team discussions separate from one another and not to interact at other times. Team ratings and comments on the health services' EQuIP performances would be compared. Observations and interviews with individual team members and the teams as a whole would be undertaken.

Secondly, inter- and intra-rater surveyor reliability will be examined using scenario-rating exercises. This would be done at ACHS surveyor training sessions. Surveyors would be asked to consider de-identified case studies individually and then as a member of an accreditation team, documenting their decisions at each point.

Thirdly, separate focus groups of surveyors would be held to explore their experiences of team-work and decision making processes. Such information would help explain any differences or similarities in reliability and consistency between individuals and teams. The groups would be conducted when the participants meet on a state by state basis around Australia for their yearly training conducted by the ACHS. Participants would be asked to volunteer for the focus groups.

#### Study 4: Prospective and retrospective study of the organisational influence of accreditation surveyors

Data relating to the ACHS accredited health services in Australia would be analysed to determine whether those with and without multiple, experienced accreditation surveyors have different performance profiles. Potential confounding factors such as health service size and casemix would be controlled for in the analyses. Up to four health services from study 1 would be randomly selected for in-depth prospective case study, involving fieldwork across the sites and qualitative analyses of surveyor influence using network influence theory [64].

## Discussion

Accreditation is a cornerstone of the safety and quality programs of many health care systems but it consumes considerable resources. We know little about its effectiveness beyond individual settings (through case study [42] or attitudinal data [37]). We argue that a program of research such as that proposed is required in order to provide research evidence regarding the relationships between clinical indicator performance, organisational culture, consumer participation and performance on accreditation standards, and to provide a basis for identifying strategies for improving health care delivery and informing policy. Without such a research approach, we run the risk of continuing to conduct expensive system-wide initiatives such as accreditation programs without an evidence base. For example, we need to reach an informed view as to whether accreditation, as it presently stands, should continue to be supported, or whether alternative methods or approaches to stimulate continuous organisational improvement should be considered. The design we propose would provide research findings which would be a pointer to questions of this type.

Although we have used EQuIP as our exemplar because of its utility and our knowledge of the Australian context, EQuIP can readily be substituted by another health system's accreditation process. This is a key international research problem because it relates to how well health sector organisations perform. This is a core constituent in whether, and the extent to which, safety prevention and early warning detection processes for health sector organisations can be realised. Through illuminating the processes by which organisational performance can be improved via accreditation mechanisms, we would be in a position to observe how the safety and quality of health care can be enhanced.

While this type of design has health care industry implications, it can be transferred to other professionalised industries eg education, law, accounting and management consulting, and potentially realise large-scale benefits to individual organisations and across industries. Furthermore, understanding organisational behaviour is now recognised as a significant issue in public health. It has come to light in recent times that despite the plethora of emerging evidence concerning clinical practice, the adoption of such evidence has been slow. For example, it has been suggested that organisational behaviour may be a factor which can either facilitate or obstruct adoption of evidence-based practice [65]. Thus, in order to propagate evidence-based practice and harmonise clinical performance indicators across the health sector so that benchmarking is possible, the institutional behaviours which facilitate or obstruct these processes need to be identified and illuminated. This type of research design is expected to contribute to our understanding of these forces.

## Conclusion

Accreditation, an international phenomenon, is found across different industries, and involves examining a range of processes and variables within organisations. While many claims are made about the benefits of accreditation processes, empirical evidence to sustain many such claims is currently lacking. Researching the impact of accreditation on individual and organisational performance is an important undertaking. There are many different accreditation systems. However, it makes sense to examine a well-developed and widely-used system in a rigorous research project such as the one outlined. Internationally, the research proposed would be highly relevant to the knowledge base on accreditation applicable across various industries and organisations.

This proposed research project has been designed in response to questions that the ACHS, customers of the accreditation services and public and private funders of health care have had for many years about the credibility, reliability and cost-effectiveness of accreditation. The results from research of this nature aim to illuminate, and possibly challenge, long-held beliefs and established processes of accreditation bodies. Understanding, for example, which organisational characteristics are positively associated with performance, or whether having consumers of the service as partners in planning, policy development and evaluation can improve outcomes is important. Similarly, assessing which factors contribute to inter-rater reliability and understanding how attitudes and behaviours of surveyors contribute to an effective system will influence the choice and training of surveyors.

The research design presented is a multi-method, multi-disciplinary, multi-level collaborative one that reflects the complex nature of the issues under consideration. In detailing our program of research prior to commencement we aim to stimulate debate about both the role of accreditation in national health care safety and quality programs and the most effective ways to study its impact.

## Abbreviations

ACHS: Australian Council on Healthcare Standards

ISO: International Standards Organization

EQuIP: Evaluation and Quality Improvement Program

## Competing interests

This research is funded by the Australian Research Council as a Linkage project which brings together industry partners [represented by MP] and academics [led by JB] to undertake partnership research. While no-one has any financial or non-financial competing interests, these kinds of research partnerships require the development of appropriate safeguards, including a clear understanding of roles, responsibilities and the arm's length nature of academic researchers. There is an acceptance of the industry partners that results may differ from their expectations and be disadvantageous to their interests, but nevertheless publication will be pursued.

## Authors' contributions

JB, RI, JW, BR, SR, MP and CJ are chief or partner investigators on the grant and made substantial contributions to the conception and design of the project and this manuscript. SN provided expertise and participated in the consumer participation component and design. DG, JN and MR helped draft the manuscript as did RG, who also provided statistical advice. All authors read and approved the final manuscript.

## Pre-publication history

The pre-publication history for this paper can be accessed here:
